# Safety of biologics and Janus kinase inhibitors in inflammatory bowel disease patients with low cardiovascular risk

**DOI:** 10.1093/crocol/otag039

**Published:** 2026-05-06

**Authors:** Paraj Patel, Omair Iqbal, Divyanshu Mohananey, Saffia Bajwa, Masoud Khani, Jake Luo, Daniel J Stein, Gursimran S Kochhar, Jana G Hashash, Emad Mansoor, Raymond K Cross, Preetika Sinh

**Affiliations:** Division of Gastroenterology and Hepatology, Medical College of Wisconsin, Milwaukee, WI, United States; Medical College of Wisconsin, Milwaukee, WI, United States; Medical College of Wisconsin Cardiovascular Center, Medical College of Wisconsin, Milwaukee, WI, United States; Department of Medicine, Medical College of Wisconsin, Milwaukee, WI, United States; Health Informatics Program, Zilber School of Public Health, University of Wisconsin-Milwaukee, Milwaukee, WI, United States; Health Informatics Program, Zilber School of Public Health, University of Wisconsin-Milwaukee, Milwaukee, WI, United States; Division of Gastroenterology and Hepatology, Medical College of Wisconsin, Milwaukee, WI, United States; Department of Gastroenterology and Hepatology, Allegheny Health Network, Pittsburgh, PA, United States; Department of Gastroenterology and Hepatology, Mayo Clinic in Florida, Jacksonville, FL, United States; Division of Gastroenterology and Liver Disease, University Hospitals Cleveland Medical Center, Case Western Reserve University, Cleveland, OH, United States; Department of Medicine, Division of Gastroenterology and Hepatology, University of Maryland School of Medicine, Baltimore, MD, United States; Division of Gastroenterology and Hepatology, Medical College of Wisconsin, Milwaukee, WI, United States

**Keywords:** inflammatory bowel disease, outcomes research, biologics, inflammation

## Abstract

**Background:**

Due to underlying chronic inflammation, inflammatory bowel disease (IBD) is associated with an increased risk of major adverse cardiovascular events (MACE). There is limited data on the role of cardiovascular (CV) risk factors and IBD medications on MACE. We aim to determine the association between MACE and IBD medications in a large national cohort of IBD patients with low baseline CV risk.

**Methods:**

We conducted a retrospective cohort study between January 1, 2015 and November 15, 2023 using the TriNetX database. Using logistic regression analysis, IBD patients exposed to biologics and small molecules were assessed for outcomes of individual and composite MACE (myocardial infarction [MI], ischemic stroke, percutaneous coronary intervention [PCI]/coronary artery bypass graft [CABG]). Outcomes were adjusted for demographics, non-advanced IBD therapies, statin use, CV risk factors, and prior history of coronary artery disease (CAD).

**Results:**

We identified 113 729 IBD patients with an average age of 42 years and low prevalence of CV risk factors (5%-6%). Anti-TNF therapy was associated with a statistically significant decreased risk of composite MACE (adjusted OR [aOR] 0.795, 95% CI, 0.670-0.930, *P* = .005) and PCI/CABG (aOR 0.56, 95% CI, 0.274-0.562, *P* = .002), and anti-integrin therapy was associated with a decreased risk of composite MACE (aOR 0.76, 95% CI, 0.580-0.980, *P* = .035). Anti-IL12/23 and JAK inhibitors showed no increased risk of MACE.

**Conclusions:**

In a cohort of young IBD patients with low CV risk, use of anti-TNF and anti-integrins is associated with a decreased risk of MACE, and there was no increased risk of MACE with JAK inhibitors.

## Graphical Abstract

**Figure:**
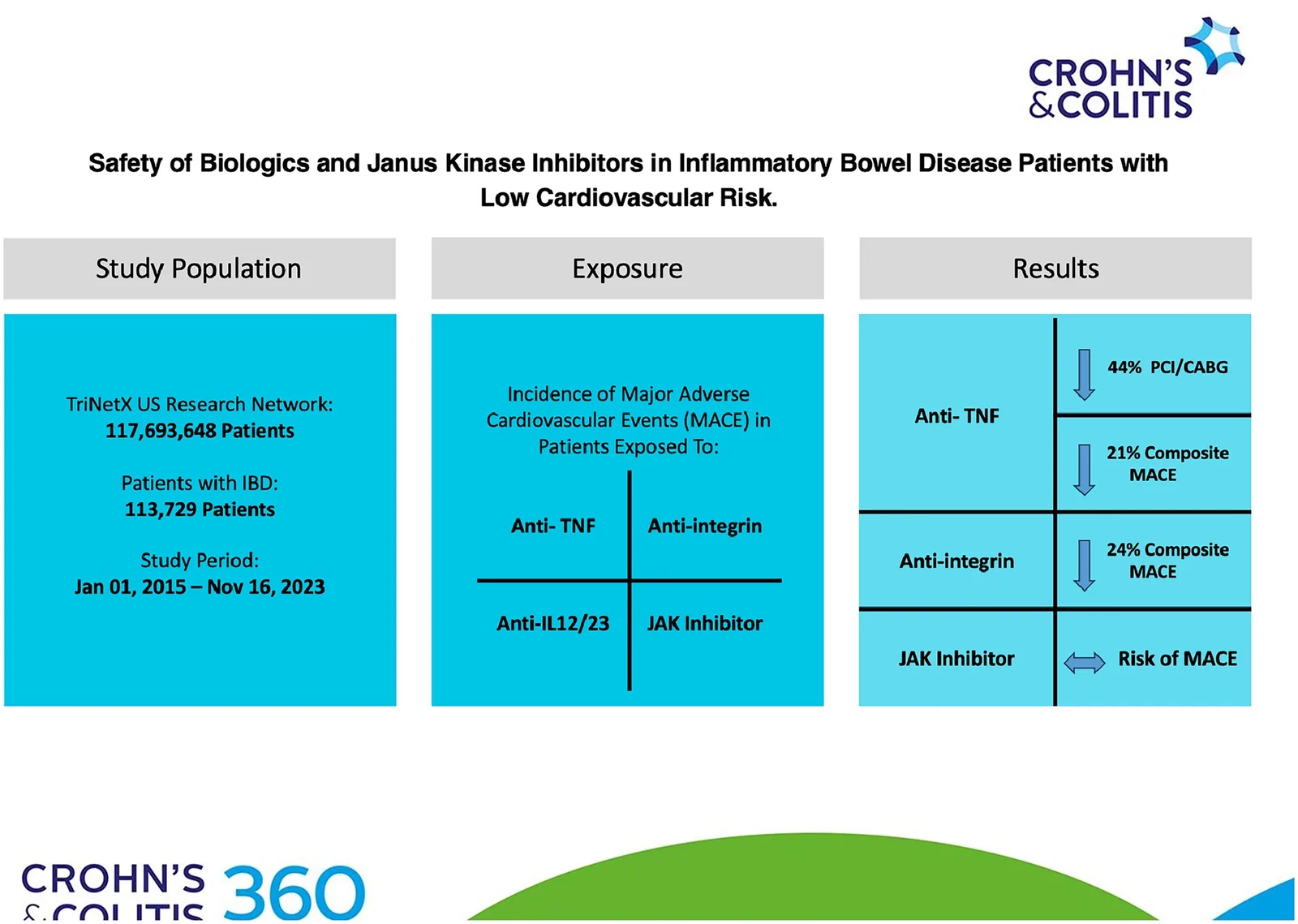

Key Points What is already known?

- Patients with IBD are at an increased risk of major adverse cardiovascular events (MACE) and data regarding the CV implications of various IBD medications is conflicting.

What is new here?

- Anti-TNF and anti-integrin medications are associated with a decreased risk of MACE while JAK inhibitors are not associated with any significant increased risk of MACE in young IBD patients with low baseline cardiovascular risk.

How can this study help patient care?

- Our analysis showed that the use of anti-TNF and anti-integrin medications in young IBD patients with low CV risk was associated with favorable cardiovascular outcomes while JAK inhibitors did not confer any increased cardiovascular risk.

## Introduction

Inflammatory bowel disease (IBD) is associated with a 2-to-4-fold increased risk of atherosclerotic cardiovascular disease (ASCVD) like myocardial infarction (MI), ischemic stroke, and heart failure (HF).^1–3^ In 2019, there were approximately 4.9 million cases of IBD worldwide and 3 million cases in the United States (US).^4^ ASCVD remains the leading cause of mortality in the US.^5^ ASCVD in IBD does not correlate well with traditional cardiovascular (CV) risk factors like hyperlipidemia (HLD), obesity, and advanced age.^1^^,^^2^ Non-traditional risk factors like female gender, younger age, and IBD disease activity have been associated with an increased risk of ASCVD events in IBD, suggesting that the underlying chronic inflammation may increase CV risk.^6–8^

Medications used to treat IBD like biologics (anti-tumor necrosis factor [TNF], anti-interleukin [IL] 12/23, anti-integrin) and small molecules (Janus kinase [JAK] inhibitors, sphingosine-1-phosphate [S1P] receptor modulators) decrease inflammation; however, it is unclear how these medications affect ASCVD.^8^ Prior studies have shown a decreased risk of ASCVD and mortality with the use of anti-TNF medications like infliximab and adalimumab.^9^ However, the ORAL surveillance study in rheumatoid arthritis (RA) patients showed that tofacitinib, a JAK inhibitor, was linked to an increased risk of ASCVD events.^10^ Interestingly, a post-hoc analysis of this trial revealed that this increased risk was most significant in patients with a prior history of ASCVD (prior coronary artery disease [CAD], prior ischemic stroke, peripheral arterial disease).^11^ Furthermore, real-world studies and data from drug trials across multiple indications for chronic inflammatory disorders, including IBD, have not revealed an increased risk of ASCVD with tofacitinib.^8^ Limited data from clinical trials of the more selective JAK inhibitor upadacitinib has ,not demonstrated an increased risk of ASCVD events in patients with IBD.^12^ These differences in CV risk amongst various IBD medications are clinically relevant, as both IBD and ASCVD may co-exist in the same patient which would necessitate the use of a medication with a favorable CV risk profile. The outcomes have not been stratified by age or baseline CV risk factors in these studies to determine safety in younger patients with low CV risk. This includes patients < 65 years of age without a history of ASCVD and low prevalence of traditional CV risk factors. The significance of CV risk in these low-risk patients is not emphasized in real-world studies because these trials are designed to be representative of the broader population.

Overall, there is limited data regarding ASCVD outcomes with specific IBD medications. Given patients with IBD are at an increased risk of ASCVD, it is vital to investigate medication-specific CV risk profiles. This would allow for a more tailored approach to manage patients with IBD across a wide spectrum of CV risk. We aim to determine the association between ASCVD and IBD medications in a large national cohort of IBD patients with low CV risk. We hypothesized that exposure to specific IBD medications is associated with a differential risk of ASCVD outcomes in patients with IBD.

## Methods

### Data source

We conducted a retrospective cohort study using the TriNetX database from January 1, 2015 to November 15, 2023. The TriNetX database provides a comprehensive dataset of de-identified patient records and ensures a broad and representative sample from 80 health care organizations (HCO) across the US with data from more than 40 million patients.

### Study population

IBD patients were identified using International Classification of Diseases (ICD-10-CM) codes for Ulcerative Colitis (UC, K51) or Crohn’s disease (CD, K50).^13^ For each patient, we recorded the index date of IBD diagnosis to establish a baseline. This critical step ensured that subsequent analyses accounted for the time between the initial onset of IBD and CV events. As shown in Table S1, inclusion criteria to identify patients with IBD were determined based on ICD-10-CM codes for either UC or CD with at least one corresponding RxNorm code for IBD medications like mesalamine, budesonide, thiopurines, methotrexate, and biologic medications. This previously validated and stringent inclusion criterion increases the reliability of using ICD-10-CM codes to detect a diagnosis of IBD and confers a positive predictive value (PPV) of >80% and specificity of >85% for the diagnosis of IBD in the TriNetx database.^13^ Patients with IBD unspecified (IBDU) were excluded due to the lack of specificity of this diagnosis in the TriNetX database. Patients with conditions that are known to increase ASCVD risk as outlined in Table S2 (familial hypercholesterolemia, celiac disease, other chronic inflammatory conditions [RA, psoriasis, psoriatic arthritis, calcium pyrophosphate deposition arthropathy, lupus, ankylosing spondylitis], HIV, and malignancy) were excluded from the study using their corresponding ICD-10-CM codes.^13^

### Data collection

The raw data was extracted by the research team directly from the TriNetX database and sent to the biostatisticians for analysis. Table 1 shows demographics, IBD type, CV risk factors, statin use, and IBD medications were collected.

**Table 1:** Baseline demographic data for inflammatory bowel disease (IBD) patients on biologic and small molecule inhibitor medications.

Medication	Anti-TNF	Anti-integrin	IL-12/23	JAK
**Patients (*N*)**	19 890	5497	5822	1087
**Age, mean (years)** **(25th, 75th percentile)**	41.8 (27.9, 52.9)	46.2 (31.9, 58.9)	43.0 (29.9, 53.9)	42.2 (29.9, 52.9)

	***N* (%)**	***N* (%)**	***N* (%)**	***N* (%)**

**Sex**				
**Male**	10 129 (50.93)	2581 (46.95)	2655 (45.60)	593 (54.55)
**Female**	9534 (47.93)	2785 (50.66)	3097 (53.19)	484 (44.53)
**Race**				
**White**	14 739 (74.10)	4151 (75.51)	4510 (77.46)	845 (77.74)
**Black or African American**	1894 (9.52)	344 (6.26)	444 (7.63)	66 (6.07)
**Asian**	497 (2.50)	203 (3.69)	145 (2.49)	38 (3.50)
**Unknown and others**	2760 (13.88)	799 (11.53)	723 (12.41)	138 (12.69)
**Type of IBD**				
**Crohn’s disease**	14 581 (73.31)	3057 (55.61)	4907 (84.28)	346 (31.83)
**Ulcerative colitis**	5122 (25.75)	2407 (43.79)	897 (15.41)	736 (67.71)
**Cardiovascular risk factors**				
**Total**	1165 (5.86)	433 (7.88)	321 (5.51)	64 (5.89)
**Hypertension**	724 (3.64)	261 (4.75)	183 (3.14)	43 (3.96)
**Hyperlipidemia**	487 (2.45)	211 (3.84)	133 (2.28)	32 (2.94)
**Type 2 diabetes**	968 (4.87)	406 (7.39)	318 (5.46)	58 (5.34
**Body mass index >35**	17 (0.09)	10 (0.18)	4 (0.07)	1 (0.09)
**Chronic kidney disease**	107 (0.54)	44 (0.80)	24 (0.41)	2 (0.18)
**Nicotine dependence**	1825 (9.18)	506 (9.21)	644 (11.06)	80 (7.36)
**Alcohol abuse**	874 (4.39)	176 (3.20)	183 (3.14)	20 (1.84)
**Chronic lung disease**	78 (0.39)	17 (0.31)	20 (0.34)	1 (0.09)
**Family history of coronary artery disease**	91 (0.46)	33 (0.60)	15 (0.26)	6 (0.55)
**Prior coronary artery disease^a^**	287 (1.40)	128 (2.30)	77 (1.32)	9 (0.83)
**Cardiovascular medication use**				
**Statin**	1305 (6.56)	442 (8.04)	386 (6.63)	111 (10.21)
**IBD non-biologic and small molecule medication use**				
**Non-advanced therapies^b^**	9340 (46.96)	2730 (49.66)	3056 (52.49)	829 (76.26)
**Immunomodulators^c^**	5151 (25.90)	1244 (22.63)	1575 (27.05)	297 (27.32)

a Prior coronary artery disease includes chronic ischemic heart disease, prior myocardial infarction, prior CABG/PCI, prior ischemic stroke, peripheral arterial disease, carotid artery disease.

b Non-advanced therapies include mesalamine, prednisone, and budesonide.

c Immunomodulators include azathioprine, mercaptopurine, and methotrexate.

### CV risk factors

To identify CV risk factors, we used ICD-10-CM codes as outlined in Table S2 corresponding to hypertension, HLD, diabetes mellitus, obesity (defined as BMI > 35), nicotine dependence, alcohol abuse, chronic kidney disease, chronic lower respiratory disease, and family history of CAD. Based on the data from post hoc analysis of the ORAL surveillance study in RA patients, which showed that prior CAD was an important factor in predicting CV outcomes, we included prior CAD as a CV risk factor in this study. As outlined in Table S2 prior CAD was defined as chronic ischemic heart disease, peripheral arterial disease, CAD, MI, ischemic stroke, and PCI/CABG prior to the IBD index date. Data on CV risk factors was collected at the time of IBD diagnosis and not at the time of the first ASCVD event. This ensured that the identified risk factors preceded the IBD diagnosis, thereby avoiding potential bias in assessing their impact on ASCVD outcomes. Considering that the study population was relatively young, this resulted in an IBD cohort with a low baseline prevalence of CV risk factors, which was also the intent of the study. The aim of this study was to assess ASCVD outcomes in patients exposed to biologics and small molecules without significant prior CV risk factors, contrary to the study population of the ORAL SURVEILLANCE study (RA patients > 50 years of age with at least one CV risk factor). ASCVD outcomes in relatively high CV risk and older population have been recently published.^14^

### Medication exposure

Patients were categorized based on their exposure to different IBD medications, including non-advanced therapies (5-aminosalicylates, oral steroids, immunomodulators [IM–azathioprine, mercaptopurine, methotrexate]), biologics (anti-TNF, anti-integrin, anti-IL12/23), and small molecules (JAK inhibitors, S1P modulators). Medication exposure was determined by identifying patients who had received these therapies and recording the use of medication after the index IBD diagnosis date. Data on medications in TriNetx is based on RxNorm prescriptions; it is not based on prescription fills, patient reports, or administrative data. To identify the duration of a medication, we must assume that (1) when a patient starts a new medication, it supplants the prior medication they were on (ie a patient on an anti-TNF agent stops this medication and starts a JAK inhibitor), and/or (2) the patient stopped taking the medication after a certain duration eg 1 year after the last prescription. Hence, data on duration of medications and combination medications was not reported due to the risk of introducing a potential source of error in the study.

### Disease activity assessment

IBD disease activity assessment was not included as a variable given the lack of a validated definition of IBD disease activity within the TriNetX database. The number of patients who had fecal calprotectin levels (stool inflammation marker) drawn during the study period was relatively low (148 total), and hence, analysis was not performed using this test as a marker for inflammation. Disease activity assessment is an important consideration when evaluating CV risk as more active disease is associated with an increased risk of myocardial infarction, stroke, and death.

### Study outcomes

The primary objective was to assess the incidence of major adverse cardiovascular events (MACE) like MI, ischemic stroke, PCI/CABG, and a composite measure of MACE that combined these outcomes. Mortality was not included in the outcomes due to lack of specificity for CV-related mortality in the TriNetx database. Primary outcomes were assessed using relevant ICD-10 and current procedural terminology (CPT) procedure codes provided in Table S2.^15^^,^^16^ Diagnosis and procedure records were filtered to identify events after the index IBD diagnosis date. This step ensured that the outcomes were after onset of IBD, allowing for a clear temporal relationship. Binary indicator variables for each outcome were added to the patient data. This step ensured that the outcomes were after onset of IBD, allowing for a clear temporal relationship. Binary indicator variables for each outcome were added to the patient data.

### Statistical analysis

This study utilized Python and the statsmodels package for data processing and statistical analysis. We employed logistic regression models to evaluate the association between IBD medications and MACE outcomes as our primary objective was to assess outcomes during a pre-specified study period, not time-to-event analysis for which a Cox proportional hazards model would be more appropriate. The models included both unadjusted and adjusted analyses. All outcomes were adjusted for age, gender, CV risk factors, prior CAD, use of non-advanced therapies, use of statins, and use of IM to limit the effects of confounding variables. The unadjusted models provided an initial assessment of the associations while the adjusted models isolated the effect of IBD therapies on the primary outcomes with limited influence of confounding variables. Each ASCVD risk factor was entered into the regression model as an individual covariate rather than as a composite yes/no variable. Modeling them separately avoids masking the differential contribution of major ASCVD risk factors (such as hypertension, diabetes, dyslipidemia, smoking, and age) compared with risk-enhancing factors (such as chronic kidney disease, chronic lung disease, alcohol use, or family history of CAD). Odds ratios (OR) with 95% confidence intervals (CIs) were calculated for each medication category, providing a measure of the relative risk associated with different treatments. This statistical approach allowed us to quantify the impact of IBD medications on ASCVD outcomes while accounting for the influence of other relevant factors.

## Results

### Demographic data

We identified 113 729 patients with IBD between January 2015 and November 2023. Most patients were on biologic therapy (anti-TNF [n = 19 890], anti-integrin [n = 5497], anti-IL 12/23 [n = 5822], JAK inhibitors [n = 1087, 743 tofacitinib, 392 upadacitinib]). A total of 130 patients exposed to S1P modulators were identified but not included in the analysis due to the small number of patients. As outlined in Table 1, the average age of patients exposed to biologic therapy was 43.7 years and on JAK inhibitors was 42.2 years. There were no significant differences in gender across the IBD cohort, and most patients were Caucasian (76%), as outlined in Table 1. Most patients exposed to anti-TNF therapy (73.3% for CD vs 25.8% for UC), anti-integrin therapy (55.6% for CD vs 43.8% UC), and anti-IL12/23 therapy (84.3% for CD vs 15.4% UC) had CD. Most patients exposed to JAK inhibitor therapy had UC (67.7% for UC vs 31.8% CD) as expected since tofacitinib (one of the two JAK inhibitors) is only approved for use in UC patients in the US. Table 1 shows there was an overall low prevalence of CV risk factors (5.5%-7.9%) and prior history of CAD (0.8%-2.3%) in the IBD cohort.

### Incidence of MACE

The incidence of MACE by IBD subtype: The incidence of MI was 429/3365 (0.80%) for CD and 395/34 907 (1.14%) for UC. The incidence of ischemic stroke was 486/53 365 (0.91%) for CD and 450/34 507 (1.30%) for UC. The incidence of PCI/CABG was 197/53 365 (0.37%) for CD and 244/34 507 (0.71%) for UC. The incidence of composite MACE was 1013/53 365 (1.90%) for CD and 992/34 507 (2.87%) for UC.

Incidence of MACE by type of IBD medication: The overall incidence of CV outcomes by drug class was low. For anti-TNF medications, the incidence of MI was 81/19 890 (0.41%), ischemic stroke was 94/19 890 (0.47%), PCI/CABG was 33/19 890 (0.17%), and composite MACE was 192/19 890 (0.97%). For anti-integrin medications, the incidence of MI was 25/5497 (0.45%), ischemic stroke was 30/5497 (0.55%), PCI/CABG was 21/5497 (0.38%), and composite MACE was 64/5497 (1.16%). For anti-IL12/23 medications, the incidence of MI was 25/5822 (0.43%), ischemic stroke was 29/5822 (0.50%), PCI/CABG was 11/5822 (0.19%), and composite MACE was 62/5822 (1.06%). For JAK inhibitors, the incidence of MI was 3/1087 (0.28%), ischemic stroke was 4/1087 (0.37%), PCI/CABG was 3/1087 (0.28%), and composite MACE was 8/1087 (0.74%).

The incidence of MACE in patients with IBD exposed to IM (total N = 13 063) was 78/13 063 (0.60%) for MI, 98/13 063 (0.75%) for ischemic stroke, 44/13 063 (0.34%) for PCI/CABG, and 192/13 063 (1.47%) for composite MACE. Logistic regression analysis for outcomes was not performed since it would have been difficult to interpret the actual effect of the drug due to lack of data on overlap with other IBD medications.

### Time to event for MACE outcomes

The time to event of each MACE outcome after the diagnosis of IBD was 2.36 years (25th and 75th interquartile range [IQR] 0.24, 3.66) for MI, 2.61 years (IQR 0.36, 3.78) for ischemic stroke, 2.69 years (IQR 0.08, 3.66) for PCI/CABG, and 2.44 years (IQR 0.24, 3.59) for composite MACE. Results of the incidence of MACE outcomes according to IBD subtype (UC and Crohn’s disease).

### Risk of MACE with IBD medications

**Figure 1:**
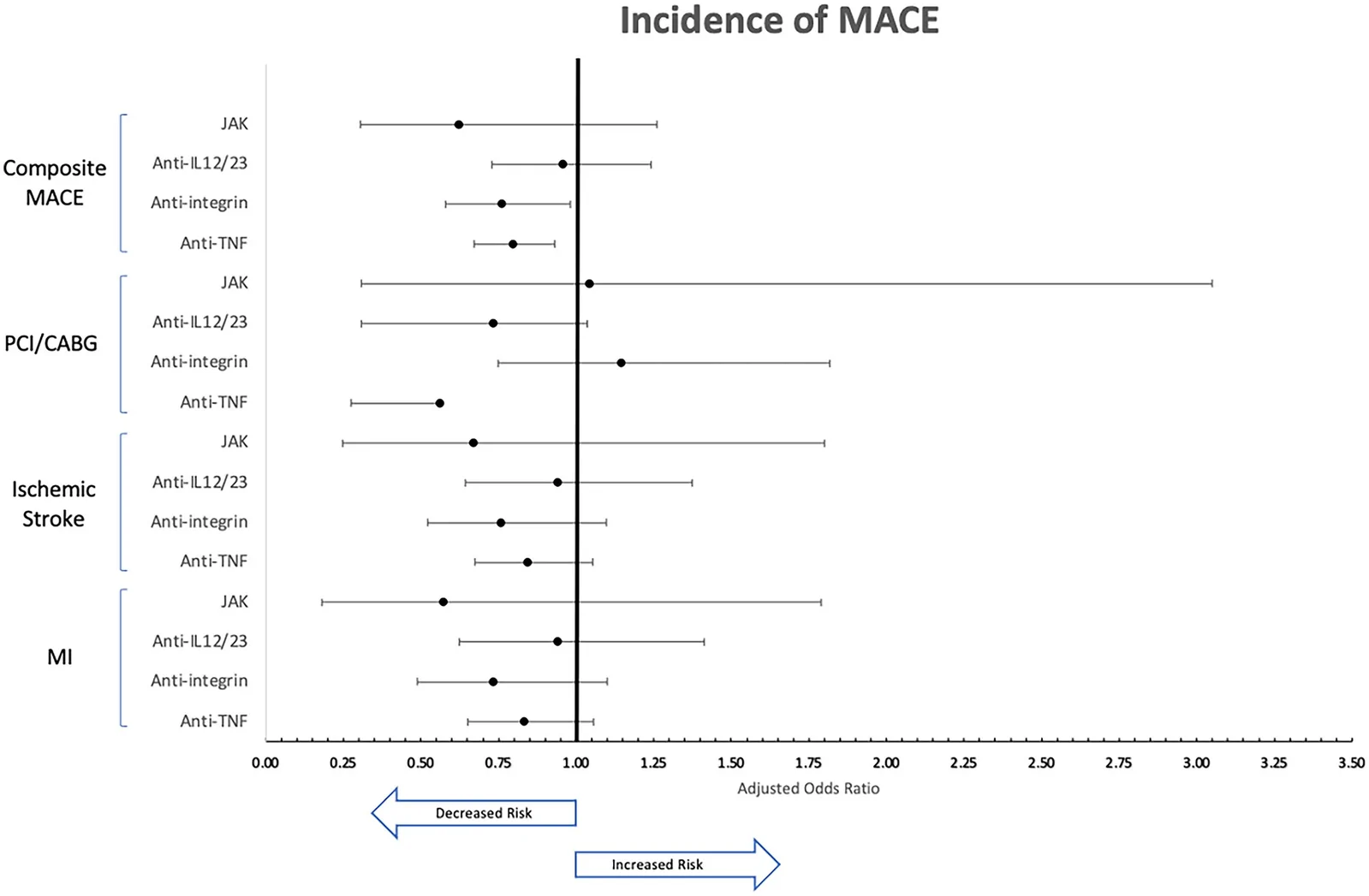
Forest plot with adjusted odd ratios of major adverse cardiovascular events by class of biologic and small molecule medications.

Anti-TNF therapy was associated with a decreased risk of MI (OR 0.539, 95% CI, 0.427-0.679, *P* < .0001), ischemic stroke (OR 0.549, 95% CI, 0.443-0.681, *P* < .0001), PCI/CABG (OR 0.393, 95% CI, 0.274-0.562, *P* < .0001), and composite MACE (OR 0.518, 95% CI, 0.445-0.602, *P* < .0001) as shown in Table 2. After adjusting for confounding variables (age, sex, cardiovascular risk factors, prior CAD and use of non-advanced therapies, statins, and immunomodulators), anti-TNF therapy showed a statistically significant risk reduction in PCI/CABG (aOR 0.559, 95% CI, 0.274-0.562, *P* = .002) and composite MACE (aOR 0.795, 95% CI, 0.670-0.930, *P* = .005) as outlined in Table 3 and Figure 1.

**Table 2:** Unadjusted odds ratios of major adverse cardiovascular events across different classes of biologic and small molecule medications.

		MI				Ischemic stroke				PCI/CABG				Composite MACE			
Medication class	*N*	No. of events	Unadjusted OR	*P*-value	95% CI	No. of events	Unadjusted OR	*P*-value	95% CI	No. of events	Unadjusted OR	*P*-value	95% CI	No. of events	Unadjusted OR	*P*-value	95% CI
**Anti-TNF**	19 890	81	**0.539**	**<.0001**	[0.427, 0.679]	94	**0.549**	**<.0001**	[0.443, 0.681]	33	**0.393**	**<.0001**	[0.274, 0.562]	192	**0.518**	**<.0001**	[0.445, 0.602]
**Anti-integrin**	5497	25	0.707	.090	[0.473, 1.055]	30	0.742	.111	[0.514, 1.071]	21	1.167	.495	[0.749, 1.816]	64	**0.743**	**.021**	[0.57, 0.955]
**Anti-IL12/23**	5822	25	0.699	.082	[0.467, 1.046]	29	0.082	.068	[0.485, 1.026]	11	0.565	.065	[0.308, 1.035]	62	**0.711**	**.009**	[0.549, 0.91]
**JAK**	1087	3	0.513	.251	[0.164, 1.603]	4	0.585	.288	[0.217, 1.571]	3	0.973	.962	[0.309, 3.05]	8	0.554	.098	[0.274, 1.115]

Abbreviations: MI = myocardial infarction; PCI/CABG = percutaneous coronary intervention/coronary artery bypass graft; MACE = major adverse cardiovascular events; TNF = tumor necrosis factor. **Bold** = statistically significant.

**Table 3:** Adjusted odds ratios of major adverse cardiovascular events across different classes of biologic and small molecule medications.

		MI				Ischemic stroke				PCI/CABG				Composite MACE			
Medication class	*N*	No. of events	Adjusted OR	*P*-value	95% CI	No. of events	Adjusted OR	*P*-value	95% CI	No. of events	Adjusted OR	*P*-value	95% CI	No. of events	Adjusted OR	*P*-value	95% CI
**Anti-TNF**	19 890	81	0.830	.130	[0.652, 1.056]	94	0.842	.134	[0.673, 1.054]	33	**0.559**	**.002**	[0.274, 0.562]	192	**0.795**	**.005**	[0.67, 0.93]
**Anti-integrin**	5497	25	0.733	.134	[0.488, 1.099]	30	0.757	.143	[0.522, 1.098]	21	1.145	.555	[0.749, 1.816]	64	**0.758**	**.035**	[0.58, 0.98]
**Anti-IL12/23**	5822	25	0.940	.765	[0.625, 1.413]	29	0.941	.752	[0.643, 1.374]	11	0.731	.314	[0.308, 1.035]	62	0.956	.739	[0.73, 1.24]
**JAK**	1087	3	0.570	.336	[0.18, 1.79]	4	0.669	0.428	[0.247, 1.80]	3	1.042	.944	[0.309, 3.05]	8	0.622	.189	[0.305, 1.26]

All outcomes were adjusted for age, sex, cardiovascular risk factors, prior coronary artery disease, use of non-advanced therapies, use of statins, and use of immunomodulators.

Abbreviations: MI = myocardial infarction; PCI/CABG = percutaneous coronary intervention/coronary artery bypass graft; MACE = major adverse cardiovascular events; TNF = tumor necrosis factor.**Bold** = statistically significant.

Anti-integrin therapy was associated with a decreased risk of MI (OR 0.707, 95% CI, 0.473-1.055, *P* = .09), ischemic stroke (OR 0.742, 95% CI, 0.514-1.071, *P* = .111), and composite MACE (OR 0.743, 95% CI, 0.570-0.955, *P* = .021) however only the risk reduction in composite MACE showed statistical significance. Table 2 shows anti-integrin therapy was associated with an increased risk of PCI/CABG (OR 1.167, 95% CI, 0.749-1.816, *P* = .495); however, this was not statistically significant. After adjusting for confounding variables, anti-integrin therapy was associated with a statistically significant decreased risk of composite MACE (aOR 0.758, 95% CI, 0.580-0.980, *P* = .035) as shown in Table 3 and Figure 1.

Anti-IL12/23 therapy was associated with a decreased risk of MI (OR 0.699, 95% CI, 0.467-1.046, *P* = .082), ischemic stroke (OR 0.706, 95% CI, 0.485-1.026, *P* = .068), PCI/CABG (OR 0.565, 95% CI, 0.308-1.035, *P* = .065), and composite MACE (OR 0.711, 95% CI, 0.549-0.910, *P* = .009); however, only the risk reduction in composite MACE reached statistical significance as shown in Table 2. After adjusting for confounding variables, there was no statistically significant association between anti-IL12/23 therapy and any of the 4 primary outcomes as shown in Table 3 and Figure 1.

JAK inhibitor therapy was associated with a decreased risk of MI (OR 0.513, 95% CI, 0.164-1.603, *P* = .251), ischemic stroke (OR 0.585, 95% CI, 0.217-1.571, *P* = .288), PCI/CABG (OR 0.973, 95% CI, 0.309-3.050, *P* = .962), and composite MACE (OR 0.554, 95% CI, 0.274-1.115, *P* = .098); however, these results did not show statistical significance as shown in Table 2. After adjusting for confounding variables, there was no statistically significant association between use of JAK inhibitors and any of the 4 primary outcomes (aOR composite MACE 0.189, 95% CI, 0.305-1.26, *P* = .189) as shown in Table 3 and Figure 1.

## Discussion

In a large national cohort of young IBD patients with a low prevalence of CV risk factors and prior CAD, anti-TNF therapy was associated with a 44% decreased risk of PCI/CABG and 21% decreased risk of composite MACE after adjusting for the presence of CV risk factors and prior CAD as shown in Table 3 and Figure 1. Use of anti-integrins was associated with a 24% decreased risk of composite MACE. JAK inhibitor therapy was not associated with an increased risk of MACE in this population. There was no significant difference in gender amongst all medication groups. The anti-TNF and anti-IL 12/23 cohorts had a preponderance of patients with CD, whereas the JAK inhibitor cohort consisted predominantly of patients with UC. Majority of patients exposed to JAK inhibitors were on tofacitinib, likely due to its initial approval for UC in the US.

It has been established that patients with IBD have an increased risk of ASCVD, presumably due to underlying chronic inflammation.^2–4^ Decreasing the inflammatory burden may decrease this risk; however, data is limited regarding the role of IBD medications for ASCVD risk reduction. Several studies have shown that anti-TNF therapy has been associated with reduction in CV risk. Analysis of a French cohort of IBD patients by Kirchgesner and colleagues showed a decreased risk of acute arterial events in those exposed to anti-TNF drugs (HR, 0.79; 95% CI, 0.66-0.95) but not to thiopurines (HR, 0.54; 95% CI, 0.82-1.05).^17^ A retrospective cohort study in the US by Lewis and colleagues showed a benefit of anti-TNF therapy compared to steroids with respect to CV mortality.^18^ While these studies suggest ASCVD risk reduction with anti-TNF therapy, they do not assess ASCVD risk reduction in newer medications used to treat IBD like anti-integrins, anti-IL12/23, and JAK inhibitors. More importantly, they do not stratify the ASCVD risk reduction according to low versus high baseline CV risk. We studied a relatively young cohort with a low baseline CV risk to assess the outcomes and safety of biologics and small molecule inhibitors. Our results indicate a risk reduction of MACE with anti-TNF therapy in this low CV risk profile cohort, which remained statistically significant (aOR 0.795, 95% CI, 0.670-0.930, *P* = .005) after adjusting for demographics, use of non-advanced therapies, use of statins, CV risk factors, and prior CAD.

Anti-integrin (eg vedolizumab) therapy has not been shown to increase risk of ASCVD in IBD patients. After adjusting for variables, anti-integrin therapy was associated with a statistically significant decreased risk of composite MACE (aOR 0.758, 95% CI, 0.580-0.980, *P* = .035).

Anti-IL12/23 inhibitors have not been associated with an increased risk of MACE in psoriasis or psoriatic arthritis, and data in IBD patients has not shown any signals of increased ASCVD risk.^19–21^ Our study shows a protective effect of this drug for composite MACE, although the results were not statistically significant.

Safety of JAK inhibitors in patients with IBD with respect to MACE is highly debated, especially in those with prior CV risk factors or CAD. In 2019, the US Food and Drug Administration (FDA) reviewed the initial results of the Oral Rheumatoid Arthritis Trial (ORAL) Surveillance Study which showed an increased risk of MACE (HR, 1.33; 95% CI, 0.91-1.94) in RA patients exposed to tofacitinib (a non-selective JAK inhibitor) when compared to anti-TNF therapy.^10^ This finding was most pronounced in patients older than 65 years and smokers. Subsequent post-hoc analysis of the ORAL Surveillance study published in 2023 only showed an increased risk of MACE in the group that already had a prior history of ASCVD.^11^ Given these findings, we analyzed the safety of JAK inhibitors in IBD patients with low baseline CV risk and low prevalence of prior CAD. We did not find any significantly increased risk of MACE with JAK inhibitors.

Strengths of our study include a large cohort of IBD patients from a national database across multiple HCO, applicability of our results to younger patients with low prevalence of CV risk factors at the time of IBD diagnosis, and statistically significant data highlighting the potential CV benefits of anti-TNF and anti-integrin therapies and the safety of JAK inhibitors in this subset of IBD patients.

There are certain limitations to our study inherent to its retrospective nature and its reliance on ICD codes for diagnosis of IBD. The IBD index date might not be the actual date of diagnosis. To increase the accuracy of the IBD diagnosis, we used a stringent and validated definition using ICD-10-CM and RxNorm medication codes, which exclude patients with indeterminate colitis and those not on IBD medications. These subgroups could have had variable risks of ASCVD events.^13^ Assessing patients only exposed to IBD medications may have led to selection bias as these patients were likely getting more health care access as compared to those not on medications or those that were not escalated to advanced therapies. We attempted to mitigate this bias by comparing patients across various IBD therapies and adjusting for non-advanced therapies in the analysis. We did not assess the duration of medication exposure or effect of switching from one mediation to another and hence the results may not reflect the true medication exposure. Another consideration is that patients who cross into institutions that are not included in the TriNetX database could have been lost to follow up. It is assumed that this number will be low and equal among groups. An important aspect to discuss is that this study did not assess IBD disease activity, which has a significant effect on ASCVD risk.^6^ Surrogate markers of IBD disease activity can be assessed (hospitalization for IBD flares, use of corticosteroids and opioids, surgical complications). Although intravenous steroids and colectomy are validated markers of severe disease in UC and have been used in prior studies, we did not include outpatient steroid or opioid data because incomplete treatment histories across health systems and inability to determine clinical indication would introduce substantial misclassification risk. Additionally, surgical codes for Crohn’s disease lack sufficient validation, limiting their use as severity proxies. Considering IBD disease activity encapsulates clinical symptoms, serum and stool inflammation markers, imaging, and endoscopic data, this variable is best studied in a prospective manner. Moreover, we only identified a small number of patients with IBD who had fecal calprotectin levels during the study period (total of 148), which limited its use as a marker for disease activity. It is also notable that there were relatively low numbers of patients exposed to JAK inhibitors in our cohort, which makes it difficult to extrapolate our findings to the more selective JAK inhibitors like upadacitinib. This study assessed multiple well-defined CV risk factors, including hypertension, HLD, nicotine dependence, and diabetes mellitus; however, the overall prevalence of these risk factors was low in this young cohort.

Results from this study suggest that in a relatively young IBD population with low baseline CV risk, biologic therapy may reduce the risk of ASCVD with the most pronounced effect in those on anti-TNF therapy. JAK inhibitors do not increase the risk of ASCVD in our cohort and can be considered relatively safe in this population.

## Supplementary Material

otag039_Supplementary_Data

## Data Availability

Data are available in the supplementary material.
