# Supplementary material for: Safety of biologics and Janus kinase inhibitors in inflammatory bowel disease patients with low cardiovascular risk
Source: Crohns Colitis 360. 2026 May 6;8(2):otag039. doi: 10.1093/crocol/otag039 (PMC13189853; doi:10.1093/crocol/otag039)
Supplement: otag039_Supplementary_Data [file otag039_supplementary_data.docx]

| **Medication Class** | **Medication** | **RxNorm Code** |
| --- | --- | --- |
| Non-Advanced | Prednisone | 8640 |
|  | Budesonide | 19831 |
|  | Mesalamine | 52582 |
|  | Sulfasalazine | 9524 |
|  | Balsalazide | 18747 |
|  | Olsalazine | 32385 |
| Immunomodulators | Azathioprine | 1256 |
|  | Mercaptopurine | 103 |
|  | Methotrexate | 6851 |
| Biologic | Infliximab | 191831 |
|  | Adalimumab | 327361 |
|  | Certolizumab | 709271 |
|  | Golimumab | 819300 |
|  | Vedolizumab | 1538097 |
|  | Ustekinumab | 847083 |
|  | Rizankinumab | 2166040 |
| Small Molecule Inhibitors | Tofacitinib | 1357536 |
|  | Upadacitinib | 2196092 |
|  | Ozanimod | 2288236 |
| Statins | Atorvastatin | 83367 |
|  | Simvastatin | 36567 |
|  | Rosuvastatin | 301542 |
|  | Pravastatin | 42463 |
|  | Lovastatin | 6472 |
|  | Pitavastatin | 861634 |

Supplementary Table 1: RxNorm codes of all medications included in the data collection and statistical analysis.

| **Cardiovascular risk factors**  Supplementary Table 2: ICD-10-CM and CPT codes for all cardiovascular risk factors, variables, and outcomes used in TriNetx. database Adjusted odds ratios of MACE events across different classes of biologic medications. | **ICD10** |
| --- | --- |
| HTN | I10-I16 |
| HLD | E78X |
| DM | E08-E13 |
| BMI 35 and over | Z68.38, Z68.4X |
| Overweight and Obesity | E66, Overweight: E66.3 |
| Nicotine dependence | F17 |
| Alcohol abuse | F10.1 |
| ﻿CKD | N18X |
| Chronic lower respiratory disease | J40-J47 |
| Family history of CAD | Z82.49 |
| **History of ASCVD** |  |
| Chronic ischemic heart disease (CAD) | I25.x |
| ﻿Prior MI | I25.2 (coded prior to index date) |
| Prior CABG/PCI | Z95.1, Z98.61 (coded prior to index date) |
| ﻿Prior TIA/Stroke | I69.9XX (coded prior to index date) |
| Peripheral arterial disease (PAD) | I73 |
| ﻿Carotid Disease | I65.2m |
| History of heart failure | I50 |
| **Disease Activity** | **CPT code** |
| Fecal Calprotectin Levels | 83993 (indicating test occurred) |
| **Outcomes after IBD index date** | **ICD10/CPT code** |
| MI | I20.0, I21.X, I24.X |
| Ischemic stroke | I619, G45X, I63, I66, G834, G9781 |
| Percutaneous Coronary Intervention (PCI) | 1021165, 92928, 92929, 92941, 1021163, 1021168, 1021166  1021167, 92973, 1012986, 92977, 92975, 1021164, 92924, 92925 |
| Coronary Artery Bypass Graft (CABG) | 1006216, 1006217, 33533, 33534, 33535, 33536, 1006207, 1006208, 33530, 1006199, 1006200, 33510, 33511, 33512, 33514, 33516 |
| Composite MACE | ICD and CPT codes combined for MI, ischemic stroke, PCI, and CABG |
